# Functional Status and All-Cause Mortality in Serious Mental Illness

**DOI:** 10.1371/journal.pone.0044613

**Published:** 2012-09-06

**Authors:** Richard D. Hayes, Chin-Kuo Chang, Andrea C. Fernandes, Aysha Begum, David To, Matthew Broadbent, Matthew Hotopf, Robert Stewart

**Affiliations:** 1 Department of Health Service and Population Research, King’s College London, Institute of Psychiatry, London, United Kingdom; 2 Biostatistics Department, King’s College London, Institute of Psychiatry, London, United Kingdom; 3 South London and Maudsley NHS Foundation Trust, London, United Kingdom; 4 Academic Department of Psychological Medicine, King’s College London, Institute of Psychiatry, London, United Kingdom; Cardiff University, United Kingdom

## Abstract

**Background:**

Serious mental illness can affect many aspects of an individual’s ability to function in daily life. The aim of this investigation was to determine if the environmental and functional status of people with serious mental illness contribute to the high mortality risk observed in this patient group.

**Methods:**

We identified cases of schizophrenia, schizoaffective and bipolar disorder aged ≥15 years in a large secondary mental healthcare case register linked to national mortality tracing. We modelled the effect of activities of daily living (ADLs), living conditions, occupational and recreational activities and relationship factors (Health of the Nation Outcome Scale [HoNOS] subscales) on all-cause mortality over a 4-year observation period (2007–10) using Cox regression.

**Results:**

We identified 6,880 SMI cases (242 deaths) in the observation period. ADL impairment was associated with an increased risk of all-cause mortality (adjusted HR 1.9; 95% CI 1.3–2.8; p = 0.001, p for trend across ADL categories = 0.001) after controlling for a broad range of covariates (including demographic factors, physical health, mental health symptoms and behaviours, socio-economic status and mental health service contact). No associations were found for the other three exposures. Stratification by age indicated that ADLs were most strongly associated with mortality in the youngest (15 to <35 years) and oldest (≥55 years) groups.

**Conclusions:**

Functional impairment in people with serious mental illness diagnoses is a marker of increased mortality risk, possibly in younger age groups as a marker of negative symptomatology.

## Introduction

Compared to the general population, individuals with serious mental illness (SMI), including schizophrenia, schizoaffective disorder and bipolar disorder, have substantially higher risk of premature mortality [1], [2], [3], [4]. This translates into a 10–20 year reduction in life expectancy, more severe in this respect than well-recognised adverse exposures such as smoking, diabetes and obesity [5]. Underlying factors however remain unclear. The mortality gap is only partially explained by suicide, accidents or violence [1], [6] and natural causes remain an important component such as death from coronary heart disease and stroke [6], [7], although these in turn are not fully explained by smoking or adverse effects of antipsychotic medication [8]. As well as research into specific outcomes and physical disorders as causal pathways, there is a need to investigate SMI subgroups to clarify whether the mortality risk is raised equally for all individuals with these diagnoses, or whether there are subgroups at particularly high risk; however, research to date has been limited and inconsistent. For example, although severity of symptomatology might be expected to exert an influence, research evidence for this has been conflicting [9], [10], [11].

SMI can impact on many aspects of an individual’s ability to function in daily life and their living environment. [12], [13], [14], [15], [16] It is possible that adverse interpersonal and lifestyle factors may contribute to the observed excess mortality [6], [17], [18], [19]. People with SMI are more likely to be socially isolated, [12], [13] living in poor housing conditions [14], may have less satisfactory diet [15], [19] and reduced physical activity [15], [20], and may experience more difficulties in managing day-to-day tasks [15], [16]. These issues are compounded by unemployment [21] or, if employed, experiences of stigma and insufficient opportunities to maintain or use intact skills resulting in disadvantaged levels of income [22].

Although previous research in SMI has explored the impact of symptoms [9], [10], [11] and risk behaviours [6], [7] we know very little about the role of environmental factors and functional status such as difficulties with activities of daily living (ADLs), living conditions, occupational/recreational activities, and relationships. However, these factors have been associated with increased risks of mortality in general population samples and other patient groups [23], [24], [25], [26], [27]. There is some evidence that not being married and lacking social support may be associated with suicidality in people with schizophrenia [28], and having no paid employment was found to increase mortality risk by 38% in a cohort admitted to an inpatient psychiatric unit [29] A recent study investigating individuals with a range of mental disorders found that clinician-appraised risk of self-neglect (but not appraised risk of suicide or violence) predicted all cause mortality, independent of physical health and to the strongest extent in the youngest age group [30]. In this investigation we examined associations between mortality and the following predictors in people with SMI: ADL impairment, social relationships, living conditions and occupational/recreational activities.

## Methods

### Ethics Statement

The Clinical Record Interactive Search (CRIS) system was approved as a dataset for secondary analysis on this basis by Oxfordshire Research Ethics Committee C (08/H0606/71).

### Setting

In the UK, there is state-wide provision of healthcare under the National Health Service (NHS), and mental health services have a close to 100% monopoly provision to defined geographic catchment areas. The South London and Maudsley NHS Foundation Trust (SLAM) provides comprehensive secondary mental healthcare to a population of approximately 1.2 million residents of four London boroughs (Lambeth, Southwark, Lewisham and Croydon) making it one of Europe’s largest mental health providers. SLAM service provision encompasses all aspects of secondary mental healthcare across all age groups including inpatient, community, general hospital liaison and forensic services. Electronic clinical records have been used comprehensively across all SLAM services since 2006, and the CRIS system, supported by the NIHR Specialist Biomedical Research Centre for Mental Health, was developed in 2008 to enable researchers to efficiently search and retrieve anonymised clinical records. There are currently over 185,000 cases represented in the CRIS system providing anonymised in-depth information. The protocol for this case register has been described in detail in an open-access publication [31].

### Inclusion Criteria

Individuals who had received an SMI diagnosis (schizophrenia [ICD-10 code: F20], schizoaffective disorder [F25] or bipolar affective disorder [F31]) during the specific observation period (from 1^st^ January 2007 to 31^st^ December 2010, inclusive) and who had been assessed by a clinician using the Health of the Nations Outcome Scale (HoNOS) [32], [33] at least once during this observation period were included in this dynamic cohort. In 1998, the HoNOS was introduced as a standard measure of patient wellbeing for application in NHS mental health services [32] and its application in all people receiving care from SLAM is a core target against which services are evaluated. HoNOS assessments were not included for individuals who were under the age of 15 at the time of assessment. Diagnoses recorded in the SLAM BRC Case Register were based on the 10^th^ edition of the World Health Organization International Classification of Diseases (ICD-10) [34].

### Main Outcome Measure

In this investigation, the outcome of interest was all-cause mortality occurring within the observation period (2007–2010, inclusive) in people with SMI. We were able to determine deaths through routine nationwide mortality tracing linked to the clinical record and therefore available for analysis, as applied in previous research [3]. Briefly, NHS numbers (a unique identifier for UK NHS medical records) for all previous and current SLAM contacts are checked monthly against the national mortality database and deaths electronically flagged on the patient record. In the UK, all death certifications are linked to NHS number, and primary and secondary healthcare providers are required by law to keep these records up to date.

### Explanatory Variables

Data from the first HoNOS questionnaire that was completed during the observation period were used to measure exposure status. The validity and feasibility of applying the HoNOS in a variety of patient groups has been assessed in a number of previous studies [32], [35], [36], [37]. The HoNOS is completed by clinicians after routine clinical assessments and is widely used as a outcome measure by English mental health services. There are 12 items in the HoNOS. Response options follow the format of 0) not a problem; l) minor problem requiring no action; 2) mild problem but definitely present; 3) moderately severe problem; 4) severe to very severe problem, with standard descriptions (available on the electronic clinical record in SLAM) for how and when to apply each level of a given item [33] **.** In this analysis the primary exposures of interest were the HoNOS items which address function across several domains: 9, 10, 11 and 12 [33]. Item 9 assesses problems with social relationships including active or passive withdrawal from social relationships, and/or non-supportive, destructive or self-damaging relationships; item 10 assesses limitations in activities of daily living such as problems with basic activities of self-care (e.g. eating, washing, dressing, toilet) as well as more complex skills such as budgeting, shopping, and use of transport; item 11 assesses suboptimal living conditions in particular whether basic necessities (heat, light, hygiene) are met; and item 12 assesses problems with occupational and recreational activities including whether there is help to cope with disabilities, and opportunities for maintaining or improving occupational and recreational skills and activities. Four further HoNOS items were included in this analysis as potential confounders. These were the items assessing a) non-accidental self injury, b) problem drinking or drug taking, c) physical illness or disability, and d) depressed mood. Due to limited numbers in some categories all HoNOS items were condensed to three response options in the analysis: 0) not a problem, 1) minor problem requiring no action, 2–4) significant problem.

Other covariates were defined from routinely completed fields on the source records. Age was calculated at the date on which each individual received their first HoNOS assessment during the observation period. Ethnic group classifications were: “White British and other white background”, “East Asian”, “South Asian”, “African, Caribbean and other black background”, and “Mixed, unknown, and others”. Relationship status was classified as being in a current relationship (cohabiting, married or civil partner) and no current relationship (divorced, civil partnership dissolved, separated, single, widowed/surviving civil partner or unknown). Employment status was classified as being in paid employment (part-time or full-time paid employment, self employed), or not in paid employment (unemployed, registered disabled, retired, full-time student including tertiary or school age, government training scheme, volunteer, not known, other). Patients were also grouped into diagnostic categories based on the first SMI diagnosis they had received during the observation period. In order to adjust for the level of mental health service involvement, we calculated the number of face-to-face contacts each patient received in a period of 60 days centred on the index HoNOS assessment. The numbers of contacts were then divided into tertiles for the analysis.

We used an area-level index of multiple deprivation to measure socioeconomic status, calculated at the level of lower super output area for the residence (LSOA) a UK address-grouping construct which contains an average of 1,500 residents. The index of multiple deprivation is derived from a range of domains applied to the area including: employment, income, education, health, barriers to housing and services, crime and the living environment. Each domain is given a specific weighting to reflect its overall importance in the calculation of this index. Moreover, each domain is made up of a number of specific indicators that reflect different aspects of the deprivation they are intended to measure. Increasing scores in the index of multiple deprivation are indicative of more severe deprivation. In this analysis, deprivation scores were divided into tertiles. The address in England that was recorded closest in time to the commencement of the observation period was used to obtain deprivation scores with a separate category for homelessness [38].

A target for clinical teams in SLAM is to perform a brief risk assessment on all active cases followed by a structured full risk assessment for those at significant risk, the latter of which includes a scale for self-neglect. However in practice only a proportion those with SMI have received a full risk assessment so the self-neglect measure was used as a covariate in a secondary analysis only. The self-neglect scale has been described in detail elsewhere [30]; briefly, binary responses for eleven tick-box items relating to self-neglect are summed to form a subscale score which was found to have reasonable internal consistency (Cronbach alpha coefficient of 0.64) [30].

### Statistical Analysis

Cox regression procedures were used to model associations between the exposures of interest (activities of daily living, living conditions, access to services and relationship factors) and all-cause mortality. Proportional hazards assumptions were tested and indicated that it was appropriate to used Cox regression procedures. For each patient, the ‘at-risk’ period commenced from the date of the patient’s first HoNOS assessment during the observation period. The censoring date was the end of the observation period (31^st^ December 2010) and the event date was the date of death during the observation period. Adjusted associations between mortality and the principal exposures of interest or potential confounders were examined. Initial models were adjusted for age, gender and diagnosis. In subsequent models we adjusted for individual or blocks of potential confounding factors: demographic characteristics, metal and physical health, contact with SLAM services and socioeconomic measures. The fully adjusted model comprised all factors listed in Table 1. Significant associations between principal exposures of interest and mortality, identified in the fully adjusted model, were then explored further by stratifying by gender, age group and diagnosis. In a secondary analysis the self-neglect risk assessment scale was included as an additional covariate.

**Table 1:** Cox regression analyses of risk factors for all cause mortality in individuals with serious mental illness (N = 6,880, 242 deaths).

Risk factors	N individuals (N deaths)	% deaths	Hazard Ratio (95% CI) adjusted for age gender& diagnosis	P value
**Demographic factors**				
** Age** (mean 41.8, SD 13.4, range 15–94years)				
15 to <35 years	2277(32)	1.4	Referent	
35 to <55 years	3454 (89)	2.6	1.8(1.2–2.8)	**0.003**
55 years and over	1149 (121)	10.5	8.6(5.8–12.7)	**<0.001**
** Gender**				
Female	3,045 (108)	3.6	Referent	
Male	3,835(134)	3.5	1.2(0.9–1.6)	0.129
** Ethnicity**				
White British or other white background	3,103(138)	4.5	Referent	
East Asian	223(6)	2.7	0.7(0.3–1.6)	0.402
South Asian	194(4)	2.1	0.5(0.2–1.3)	0.168
African, Caribbean, other black background	2,805(80)	2.9	0.8(0.6–1.0)	0.102
Mixed, unknown others	555(14)	2.5	0.9(0.5–1.6)	0.774
** Married or cohabiting**				
No	6,024(218)	3.6	Referent	
Yes	856 (24)	2.8	0.7(0.5–1.1)	0.103
**Socioeconomic factors**				
** Deprivation level in area of residence** (tertiles)				
Low levels of deprivation	2,189(84)	3.8	Referent	
Medium levels of deprivation	2,196(81)	3.7	1.0(0.8–1.4)	0.883
High levels of deprivation	2,197(64)	2.9	0.8(0.6–1.1)	0.203
Homeless	144(4)	2.8	0.8(0.3–2.1)	0.616
** Employment status**				
Not in paid employment	6,550(240)	3.7	Referent	
In paid employment	330(2)	0.6	0.3(0.1–1.1)	0.070
**Mental and physical health factors**				
** SMI Diagnosis**				
Schizophrenia (ICD10 code - F20)	4,270(170)	4.0	Referent	
Schizoaffective disorder (ICD10 code - F25)	771(28)	3.6	1.0(0.7–1.5)	0.926
Bipolar affective disorder (ICD10 code - F31)	1,839(44)	2.4	0.6(0.5–0.9)	**0.007**
** Depressed mood**				
Not a problem	3,061(105)	3.4	Referent	
Minor problems only	1,993(85)	4.3	1.4(1.1–1.9)	**0.020**
Significant problem	1,801 (50)	2.8	1.1(0.8–1.6)	0.448
** Non-accidental self-injury**				
Not a problem	6,020(209)	3.5	Referent	
Minor problem only	475(20)	4.2	1.6(1.0–2.5)	0.057
Significant problem	364(11)	3.0	1.4(0.7–2.5)	0.327
** Problem-drinking or drug taking**				
Not a problem	4,975(174)	3.5	Referent	
Minor problems only	738(28)	3.8	1.5(1.0–2.3)	0.050
Significant problem	1,084(35)	3.2	1.4(0.9–2.0)	0.091
** Physical illness or disability problems**				
Not a problem	4,313(71)	1.7	Referent	
Minor problems only	1,241(61)	4.9	2.0(1.4–2.9)	**<0.001**
Significant problem	1,292(108)	8.4	2.9(2.1–4.0)	**<0.001**
**Face-to-face contact with SLAM services** (tertiles)				
Low level of contact	2,013(61)	3.0	Referent	
Medium level of contact	2,540(89)	3.5	1.0(0.8–1.5)	0.774
High level of contact	2,327(92)	4.0	1.5(1.1–2.0)	**0.020**
**General living environment**				
** Activities of daily living (ADLs)**				
Not a problem	3,124(63)	2.0	Referent	
Minor problems only	1,628(58)	3.6	1.5(1.0–2.1)	**0.033**
Significant problem	2,074(119)	5.7	2.3(1.7–3.2)	**<0.001**
** Standard of living conditions**				
Not a problem	4,064(129)	3.2	Referent	
Minor problems only	1,261(45)	3.6	1.0(0.7–1.5)	0.799
Significant problem	1,321(61)	4.6	1.5(1.1–2.1)	**0.005**
** Occupational and recreational activities**				
Not a problem	2,916(88)	3.0	Referent	
Minor problems only	1,617(67)	4.1	1.4(1.02–1.9)	**0.033**
Significant problem	2,135(80)	3.8	1.4(1.04–1.9)	**0.025**
** Social relationships**				
Not a problem	2,515(91)	3.6	Referent	
Minor problems only	1,836(57)	3.1	0.9(0.6–1.2)	0.496
Significant problem	2,440(91)	3.7	1.2(0.9–1.6)	0.213

## Results

We identified 6,880 individuals with a primary diagnosis of schizophrenia, schizoaffective disorder or bipolar disorder who met the inclusion criteria, in whom 242 deaths occurred during the observation period. The mean follow-up period was 974.8 days (SD 412.4 days). Of these cases, approximately three quarters (74.8%) had received at least one HoNOS assessment during the observation period. Compared to those who had received this assessment, those missing the HoNOS assessment were older (mean (SD) ages at first SMI diagnosis: 51.2 (19.9) vs. 41.8 (13.4) years respectively), were more likely to have died during the observation period (9.6% vs. 3.5%), were more likely to be female (49% vs. 44%) but did not differ with regard to paid employment status. In addition there were small differences in the proportion of SMI diagnoses comparing those missing the HoNOS assessment and those receiving it (schizophrenia: 59.0% vs. 62.1%; schizoaffective disorder: 8.3% vs. 11.2%; bipolar affective disorder 32.7% vs. 26.7%).

Table 1 presents Cox regression analyses of risk factors for all-cause mortality in individuals with serious mental illness adjusting for age, gender and diagnosis. Numbers of cases and number and percentage of deaths are presented by levels of exposure variables. After controlling for age, gender and diagnosis, mortality was positively associated with ADL impairment; significant problems with standard of living conditions; and problems with occupational and recreational activities. Associations were also observed between mortality and older age, diagnosis (reduced risk in bipolar disorder compared to schizophrenia), minor problems with depressed mood, worse physical health and higher mental health service contact. A small proportion of cases included in this investigation had received different SMI diagnoses (on different occasions) during the observation period (n = 316, 4.6%). The proportion of individuals who had received different SMI diagnoses was highest among those initially diagnosed with schizoaffective disorder, (n = 75, 9.7%) and lower among those initially diagnosed with schizophrenia (n = 150, 3.5%) or bipolar disorder (n = 91, 5.0%).

Table 2 summarises multivariable analyses of associations between exposures of interest and mortality after adjusting for individual or blocks of potential confounders. A likelihood ratio test indicated that it was appropriate to use age as a continuous variable in the models. In addition, as a sensitivity analysis, we included age as a quadratic in the models but this made very little difference to the results. Associations between mortality and the standard of living conditions and occupational or recreational activities were observed after adjusting for demographic factors only; however after adjusting for both demographic and health factors, mortality was not significantly associated with living conditions, occupational and recreational activities or social relationships. By contrast, problems with ADLs remained associated with an increased risk of all-cause mortality across all models including the fully adjusted model (assessed by p value for trend across categories). Moreover, in the fully adjusted model significant ADL impairment was independently associated with mortality risk (adjusted HR 1.9; 95% CI 1.3–2.8; p = 0.001).

**Table 2:** Multivariate Cox regression analyses of functional status and environment risk factors for all-cause mortality in individuals with serious mental illness.

	Hazard Ratio (95% CI)				
General living environment risk factors	Adjusted for demographic^a^ factors	Adjusted for demographic^a^, & health factors^b^	Adjusted for demographic^a^, health^b^ & socioeconomic^c^	Adjusted for demographic^a^ health^b^ & SLAM service contact^d^	Fully adjusted^e^
**Activities of daily living (ADLs)**					
Not a problem	Referent*	Referent*	Referent*	Referent*	Referent*
Minor problems only	1.5(1.0–2.1)	1.3(0.9–1.9)	1.3(0.9–1.9)	1.3(0.9–1.9)	1.4(0.9–2.0)
Significant problem	2.3(1.7–3.2)	1.9(1.3–2.6)	1.9(1.4–2.7)	1.8(1.3–2.5)	1.9(1.3–2.8)
**Standard of living conditions**					
Not a problem	Referent*	Referent	Referent	Referent	Referent
Minor problems only	1.1(0.7–1.5)	1.0(0.7–1.4)	0.9(0.6–1.3)	0.9(0.7–1.3)	0.8(0.6–1.2)
Significant problem	1.5(1.1–2.1)	1.3(1.0–1.8)	1.4(1.0–1.9)	1.3(0.9–1.7)	1.1(0.8–1.6)
**Occupational and recreational activities**					
Not a problem	Referent*	Referent	Referent	Referent	Referent
Minor problems only	1.4(1.0–1.9)	1.3(1.0–1.8)	1.3(0.9–1.8)	1.3(0.9–1.8)	1.2(0.9–1.7)
Significant problem	1.4(1.0–1.9)	1.2(0.9–1.7)	1.2(0.8–1.6)	1.2(0.8–1.6)	0.9(0.6–1.3)
**Social relationships**					
Not a problem	Referent	Referent	Referent	Referent	Referent
Minor problems only	0.9(0.6–1.2)	0.8(0.6–1.2)	0.9(0.6–1.2)	0.8(0.6–1.2)	0.8(0.6–1.2)
Significant problem	1.2(0.9–1.6)	1.0(0.8–1.4)	1.1(0.8–1.5)	1.0(0.7–1.4)	0.9(0.6–1.3)

* P for trend <0.05.

a Demographic factors  =  age, gender, ethnicity, married or cohabiting.

b Health factors (mental & physical)  =  Serious mental illness diagnosis, depressed mood, non-accidental self-injury, problem-drinking or drug taking, physical illness or disability problems.

c Socio-economic factors  =  deprivation level in area of residence, employment status.

d SLAM service contact ** = ** face-to-face contact with any South London and Maudsley services over a period of 60 days centred on the date of diagnosis.

e Fully adjusted  =  adjusted for demographic factors, health factors socio-economic factors and SLAM service contact (as described above) and other general living environment factors appearing in this table.

In Tables 3, 4 and 5 associations between ADL impairment and all-cause mortality in individuals with serious mental illness are stratified by gender, age group and diagnosis, respectively. Significant problems with ADLs were associated with mortality in both men and women in partially (adjusted for age and diagnosis) and fully adjusted models (Table 3). After adjusting for age (as a continuous variable), gender and diagnosis, (Table 4) ADL impairment was significantly associated with mortality in the youngest (15 to <35 years) and oldest (≥55 years) age groups investigated but not in the mid age group (35 to <55 years). This same pattern occurred in fully adjusted models. However, a likelihood ratio test including interaction terms indicated that there was insufficient evidence that the relationship between ADLs and mortality varied by age group (p = 0.245). Stratifying by diagnosis (Table 5), ADL impairment was associated with mortality in all SMI groups in age and gender adjusted models. However, in fully adjusted models significant associations between ADL impairment and mortality were evident for those diagnosed with schizophrenia and schizoaffective disorder but not bipolar disorder.

**Table 3:** Association between activities of daily living (ADLs) and all-cause mortality in individuals with serious mental illness stratified by gender.

	Hazard Ratio (95% CI), P value			
Activities of daily living (ADLs)	Adjusted for age & diagnosis		Fully Adjusted*	
**Female** (N = 3020, 107 deaths)				
Not a problem with ADLs	Referent		Referent	
Minor problems only with ADLs	1.6(0.9–2.7)	0.084	1.4(0.8–2.5)	0.286
Significant problem with ADLs	2.3(1.4–3.6)	**<0.001**	1.8(1.0–3.2)	**0.046**
**Male** (N = 3804, 133 deaths)				
Not a problem with ADLs	Referent		Referent	
Minor problems only with ADLs	1.4(0.9–2.3)	0.179	1.3(0.8–2.2)	0.350
Significant problem with ADLs	2.4(1.6–3.6)	**<0.001**	2.1(1.2–3.4)	**0.005**

* Adjusted for demographic factors(age, ethnicity, married or cohabiting); mental and physical health factors (serious mental illness diagnosis, depressed mood, non-accidental self-injury, problem-drinking or drug taking, physical illness or disability problems); socio-economic factors (deprivation level in area of residence, employment status); general living environment factors (activities of daily living, standard of living conditions, occupational and recreational activities, social relationships) and SLAM service contact **(**face-to-face contact with any South London and Maudsley services over a period of 60 days centred on the date of diagnosis).

**Table 4:** Association between activities of daily living (ADLs) and all-cause mortality in individuals with serious mental illness stratified by age.

	Hazard Ratio (95% CI), P value			
Activities of daily living (ADLs)	Adjusted for age, gender & diagnosis		Fully adjusted*	
**15 to <35 years** (N = 2256, 32 deaths)				
Not a problem with ADLs	Referent		Referent	
Minor problems only with ADLs	1.3(0.5–3.3)	0.636	1.5(0.5–4.7)	0.470
Significant problem with ADLs	2.3(1.0–5.3)	**0.041**	2.8(1.0–8.0)	**0.049**
**35 to <55 years of age** (N = 3428, 89 deaths)				
Not a problem with ADLs	Referent		Referent	
Minor problems only with ADLs	1.4(0.8–2.3)	0.247	1.4(0.8–2.5)	0.256
Significant problem with ADLs	1.5(0.9–2.4)	0.117	1.3(0.7–2.4)	0.385
**55 years and over** (N = 1140, 119 deaths)				
Not a problem with ADLs	Referent		Referent	
Minor problems only with ADLs	1.7(1.0–3.1)	0.056	1.5(0.8–2.8)	0.241
Significant problem with ADLs	3.3(2.0–5.4)	**<0.001**	2.1(1.2–3.8)	**0.011**

* Adjusted for demographic factors(age, gender, ethnicity, married or cohabiting); mental and physical health factors (serious mental illness diagnosis, depressed mood, non-accidental self-injury, problem-drinking or drug taking, physical illness or disability problems); socio-economic factors (deprivation level in area of residence, employment status); general living environment factors (activities of daily living, standard of living conditions, occupational and recreational activities, social relationships) and SLAM service contact **(**face-to-face contact with any South London and Maudsley services over a period of 60 days centred on the date of diagnosis).

**Table 5:** Association between activities of daily living (ADLs) and all-cause mortality in individuals with serious mental illness stratified by diagnosis.

	Hazard Ratio (95% CI), P value			
Activities of daily living (ADLs)	Adjusted for age & gender		Fully adjusted*	
**Schizophrenia** (N = 4233, 168 deaths)				
Not a problem with ADLs	Referent		Referent	
Minor problems only with ADLs	1.4(0.9–2.2)	0.102	1.4(0.9–2.3)	0.161
Significant problem with ADLs	2.1(1.5–3.1)	**<0.001**	1.8(1.2–2.9)	**0.009**
**Schizoaffective disorder** (N = 765, 28 deaths)				
Not a problem with ADLs	Referent		Referent	
Minor problems only with ADLs	1.5(0.5–4.3)	0.452	1.9(0.6–6.2)	0.265
Significant problem with ADLs	2.9(1.2–7.2)	**0.022**	3.2(1.1–9.5)	**0.039**
**Bipolar affective disorder** (N = 1826, 44 deaths)				
Not a problem with ADLs	Referent		Referent	
Minor problems only with ADLs	1.6(0.7–3.6)	0.245	1.2(0.4–3.0)	0.765
Significant problem with ADLs	2.8(1.4–5.7)	**0.004**	2.0(0.8–4.9)	0.127

* Adjusted for demographic factors(age, gender, ethnicity, married or cohabiting); mental and physical health factors (depressed mood, non-accidental self-injury, problem-drinking or drug taking, physical illness or disability problems); socio-economic factors (deprivation level in area of residence, employment status); general living environment factors (activities of daily living, standard of living conditions, occupational and recreational activities, social relationships) and SLAM service contact **(**face-to-face contact with any South London and Maudsley services over a period of 60 days centred on the date of diagnosis).

The proportion of the cohort who had completed at least one item on the self-neglect risk scale was 60.3%. When this variable was included in a fully adjusted model (in the subsample who had completed at least one item on the self-neglect risk scale) it made little difference to the association between ADL impairment and mortality (adjusted HR 2.3; 95% CI 1.3–4.1; p = 0.006). In a sensitivity analysis we coded all those who had not received a full risk assessment as having scored zero so that these individuals would not be lost from the analytic sample but again including this variable made little difference to the association between ADL impairment and mortality (adjusted HR 2.1; 95% CI 1.4–3.0; p<0.001).

## Discussion

In this investigation, we linked all-cause mortality data to a secondary mental healthcare case register to examine whether aspects of the living environment and functional status contribute to mortality in individuals with SMI. ADL impairment was found to be associated with increased risk of mortality after adjustment for a number of covariates. Clinically significant ADL impairment was associated with mortality in both men and women and in the youngest (under 35 years) and oldest (55 years and over) age groups investigated, indicating that it wasn’t confined to the older age groups, although age interaction tests were not significant. After adjusting for demographic and health factors, mortality was not significantly associated with other general living environment factors investigated including living conditions, occupational and recreational activities or social relationships.

To our knowledge, no previous research has specifically investigated associations between functional status and mortality in individuals with SMI. Research into the impact of ADL impairment on mortality risk has almost exclusively focussed on older community samples [25], [39], and ADL impairment is clearly associated with increased age and physical illness. [40] However, these associations are likely to be of limited relevance in this cohort where ADL impairment was associated with an increased risk of mortality in those under 35 as well as among older individuals and where the association persisted after controlling for a broad range of potential confounders including age, physical health, alcohol or drug misuse, depression and non-accidental self injury. Factors underlying score distributions on this scale are likely to differ in younger and older age groups and the construct in SMI will be considered further below. There was no association between all-cause mortality and prior problems with social relationships or relationship status, although an earlier investigation reported that not being married and lacking social support were associated with suicidality in schizophrenia [28]. Although cause of death was not analyzed as an outcome in our study, there is evidence that a sizable proportion of excess mortality in people with SMI is due to natural causes, [1], [6] and specific associations with suicide are unlikely to be reflected here. Having no paid employment was found to increase risk of mortality in a previous study of individuals admitted to an inpatient psychiatric unit. [29] However, in our sample employment status was not associated with mortality in partially or fully adjusted models and deficits in occupational/recreational activities were we no longer associated with mortality after controlling for demographic and health factors, although the latter are potentially on the causal pathway between socioeconomic status and mortality so that findings after adjustment should be viewed with caution.

This investigation has a number of strengths. The sample was large and inclusive of all patients with SMI who had made contact with mental health services within a defined area over a 4-year period. SLAM is a large provider of secondary mental healthcare and a near-monopoly provider for its geographic catchment, hence our data should be representative of patients with SMI living in urban and suburban areas. In this longitudinal analysis, it was possible to draw on complete electronic clinical records of more than six thousand cases, providing the statistical power to simultaneously control for a range of potential confounders. In the UK, it is a legal requirement for primary and secondary healthcare providers to keep death records up to date. Mortality tracing in the source records system is updated monthly and is based on national certification so that only deaths occurring outside the UK are likely to have been missed.

However, there are limitations that need to be considered. We controlled for a wide range of potential confounders; however, there may still be residual confounding. In particular, medication use was not included in this analysis which may have had an impact on results, for example relating to physical consequences of long-term antipsychotic use [6]. Also, a reliable assessment of duration of illness (or chronic versus episodic illness) and level of access to basic primary medical care services were not available for this cohort. Moreover Adverse lifestyle choices (other than drinking problems), such as smoking, poor diet, and physical inactivity, may also contribute to the increased risk of mortality in individuals with SMI. [6], [17], [18], [19] Data on these variables were not available, although these are more likely to represent mediating rather than confounding factors (i.e. potentially a result of ADL impairment and therefore lying on the causal pathway between this status and mortality as an outcome). ADL impairment was assessed using a single HoNOS item, consequently it was not possible to explore the relative contributions of basic versus instrumental ADL. Also cognitive function was not assessed in this analysis. Three quarters of those with SMI had received at least one HoNOS assessment during the observation period but there were differences between those with and without this assessment. Most notably, those missing the HoNOS assessment were older and more likely to have died during the observation period, differences which may have impacted on results. It should be noted that HoNOS is not a research assessment and inter-rater variation may have reduced the apparent effect of the exposures examined. All secondary mental healthcare within the four boroughs that form the SLAM catchment is provided at no cost to consumers as part of the UK National Health Service (NHS), so the only missing mental health service contacts would be from individuals seeking exclusively private healthcare [41]. However, the characteristics of the cohort who are known to secondary care may still be influenced by levels of disadvantage or referral bias. Consequently, the generalisability of these findings is principally to secondary care rather than primary care populations.

The results of this investigation have potential implications for clinicians and researchers. There is evidence from previous research that unhealthy lifestyles and poor general health may make a major contribution to mortality among individuals with SMI [6], [17], [18], [19] including evidence from our own sample on sub-clinical illness. [11] The analyses described here highlight difficulties with ADLs as a factor defining a group at higher risk for mortality. While in older age groups, this may reflect comorbid physical frailty (although the association was independent of physical health to the extent that it was characterised here), this is less likely to be a meaningful explanation in the younger age group, where the association remained strong and significant. Functional decline is a core feature of the negative syndrome in schizophrenia and higher scores on the HoNOS ADL scale in younger people might well be indicative of this. Taken together with recent findings of self-neglect risk and increased mortality from a related cohort in this case register [30], and generally few associations between HoNOS symptom or behaviour scales and mortality [11], the overall picture is one of highest all-cause mortality risk in service users who may be least visible to clinical teams. Causal pathways require further delineation (for example, the extent to which associations are accounted for by adverse lifestyles or difficulties accessing healthcare). However, ADL impairment can be identified and rated by care-coordinators and both HoNOS and more in-depth evaluation might well provide an easily identified early warning for premature mortality and a potential point for intervention. Routine assessment of functional status among those with SMI can generate important information allowing service providers to better prioritise care. The HoNOS is widely used as a clinical outcome measure in SLAM and across English mental health services generally. In clinical settings beyond the UK other routine assessment tools (if widely available and well completed) may be used to provide similar information. In future, health management of individuals with SMI could more effectively target difficulties with ADLs to help reduce the excess mortality risk among those with SMI, although this would require further evaluation. Further research might employ mixed methods to clarify the features of ADL impairment in the context of SMI (particularly in the under 35 age group) with a view to gaining a better understanding of potential causal pathways between ADLs and mortality as well as cause specific mortality and co-existing medical illnesses.
